# Editorial: Glial Plasticity in Depression

**DOI:** 10.3389/fncel.2016.00163

**Published:** 2016-06-17

**Authors:** João F. Oliveira, Catarina A. Gomes, Sandra H. Vaz, Nuno Sousa, Luisa Pinto

**Affiliations:** ^1^Life and Health Sciences Research Institute, School of Health Sciences, University of MinhoBraga, Portugal; ^2^Life and Health Sciences Research Institute/3B's—PT Government Associate LaboratoryBraga/Guimarães, Portugal; ^3^DIGARC, Polytechnic Institute of Cávado and AveBarcelos, Portugal; ^4^Faculty of Medicine, Center for Neuroscience and Cell Biology, University of CoimbraCoimbra, Portugal; ^5^Institute for Biomedical Imaging and Life Sciences and Faculty of Medicine, University of CoimbraCoimbra, Portugal; ^6^Faculdade de Medicina, Instituto de Medicina Molecular, Universidade de LisboaLisboa, Portugal; ^7^Faculdade de Medicina, Instituto de Farmacologia e Neurociências, Universidade de LisboaLisboa, Portugal

**Keywords:** glia, depression, astrocyte, microglia, oligodendrocites

Depression is a highly prevalent disorder that poses a significant social burden to society. Despite continued advances toward the understanding of the pathophysiology of this disease, its molecular/cellular underpinnings remain elusive, which may be at the basis of the lack of effective treatment strategies. Among the different lines of research, recent literature suggests that impaired neuron and glial plasticity may be a key underlying mechanism in the precipitation of the disorder. Surprisingly, glial cells appear to be involved both in the pathophysiology of major depression and in the action of antidepressants. In particular, several works refer to alterations in the morphology and numbers of astrocytes, microglia, and oligodendrocytes in the context of depression, in human patients, and animal models of depression. These observations are linked to functional evidences, such as impairments in the cross-talk between glia and neurons, changes in the level of neurotransmitter or immunoactive substances, myelination status, and synapse formation, maintenance or elimination.

This Research Topic highlights the roles played by neurons, astrocytes, and microglia in depressive disorder(s). Polyakova et al. begin by suggesting the astrocytic S100B as a novel marker of minor depression, specifically in males, which could help to understand its pathophysiology. The study point outs the possible relevance of glial cells in the modulation of brain neuron-glia networks at least in some types of depression. Rial et al. explore the multiple interactions between glial and neuronal cells at the synaptic level, which may be impaired in depressive-like conditions. The authors address how purines may be used to restore synaptic efficacy by modulating glia-neuron bidirectional communication, possibly reverting depressive-like behaviors. Jo et al. discuss the importance of the glial-mediated immune modulation through cytokine signaling that may trigger depressive episodes, and Branchi et al. highlight that by participating actively in the modulation of the extracellular environment microglia are an integral part of brain plasticity and, therefore, appear to account directly for the precipitation of the depressive disorder. Brites and Fernandes point out that the disruption of secreted extracellular vesicles, an alternative form of neuro-glial signaling, may also underlie depressive behavior. Collectively the research indicates that these novel forms of neuro-glia communication may represent a novel approach for modulation of the brain networks and, thus, its manipulation may allow the development of autologous therapies for depression.

In addition to the implication of glia in the pathophysiology (and treatment) of depression, a number of studies associates glia-related pathways to classically accepted antidepressant mechanisms. Specifically, in this Research Topic, Di Benedetto et al. suggest a link between fluoxetine modulation of aquaporin four levels in astrocytes with consequences for astrocyte morphology and re-establishment of a functional glia-vasculature interface, which may underlie its antidepressant effect. Interestingly, the decreased levels of astrocyte-specific connexin 43 (Cx43) appear to be related with antidepressant and anti-anxiolytic phenotypes as suggested by Quesseveur et al. Moreover, the authors suggest that the inactivation of Cx43 might induce beneficial effects through an attenuation of the stress response, “avoiding” depressive symptoms. In accordance, Jeanson et al. explored the effect of antidepressants on the functional status of astrocytic connexins, showing a complex pattern of responses that links astrocytes to the mode of action of these drugs. Exploring the use of deep brain stimulation (DBS) as an alternative for treatment-resistant depression patients, Etiévant et al. discuss the beneficial role of astrocytes in the process based on the absence of a DBS-effect after pharmacological lesion of astrocytes.

Overall, the excellent reflections and novel data sets that make up this Research Topic provide evidence for a role of glial cells, namely microglia and astrocytes, in the mechanisms underlying depression and the effect of antidepressants. Collectively, the data indicates that despite the rapid advancement of the field there is still a long way to go. The study of glial cells will continue to reveal novel and more effective therapeutic mechanisms for depression and its symptomatology.

## Author contributions

All authors listed, have made substantial, direct and intellectual contribution to the work, and approved it for publication.

## Funding

JO and LP received fellowships from the Foundation for Science and Technology (FCT) and their work is funded by FCT (SFRH/BD/101298/2014 to JO and IF/01079/2014 to LP) and Bial Foundation (207/14 for JO and 427/14 for LP) projects. SV is supported by FCT (SFRH/BPD/81627/2011). CG is supported by FCT (SFRH/BPD/63013/2009). This work was co-funded by the Life and Health Sciences Research Institute (ICVS), and Northern Portugal Regional Operational Programme (NORTE 2020), under the Portugal 2020 Partnership Agreement, through the European Regional Development Fund (FEDER) (projects NORTE-01-0145-FEDER-000013 and NORTE-01-0145-FEDER-000023). This work has been also funded by FEDER funds, through the Competitiveness Factors Operational Programme (COMPETE), and by National funds, through the (FCT), under the scope of the project POCI-01-0145-FEDER-007038.

### Conflict of interest statement

The authors declare that the research was conducted in the absence of any commercial or financial relationships that could be construed as a potential conflict of interest.

